# Resources and Services for Family Caregivers in the Time of COVID-19

**DOI:** 10.1093/geroni/igab046.1247

**Published:** 2021-12-17

**Authors:** Gwen McGhan, Deirdre McCaughey, Kristin Flemons, Whitney Hindmarch

**Affiliations:** University of Calgary, Calgary, Alberta, Canada

## Abstract

COVID-19 has led to increased burden on family caregivers (FCGs) for people living with dementia (PLWD), while simultaneously limiting the resources available to them. Our study surveyed Alberta, Canada FCGs to assess their needs and generate recommendations to inform policies about care access, resources, and agency supports. We conducted a mixed methods study using a sequential triangulation design (QUANTITATIVE + qualitative). Our Community Advisory Committee was involved in all stages of study planning, execution, and dissemination. Survey results informed the qualitative data collected from focus groups with FCGs. A total of 230 FCGs participated in the survey, with an average age of 59. The average age of PLWD was 75. The majority were women (77%), 46% were spouses and 41% were adult children. Respondents reported feeling more isolated (69%), more strain (66%) and decreased quality of life (55%) compared to pre-pandemic. Resource use by FCGs decreased from an average of 5 resources pre-pandemic to 1.6 during COVID-19. Services including day programs and home care were no longer available or reconfigured, leading to greater strain and heightened need for respite, which was also unavailable. Focus groups highlighted that system navigation and accessing services during COVID-19 was overly burdensome, leaving FCGs feeling abandoned by the system. FCGs reported an increase in caregiving responsibility and less access to services resulting in PLWD experiencing a decline in wellness and function. As such: 1) resources should be consistently available for FCGs and 2) FCGs require clear, correct, and concise information about COVID-19.

